# Differential effects of Chinese high-fat dietary habits on lipid metabolism: mechanisms and health implications

**DOI:** 10.1186/s12944-020-01212-y

**Published:** 2020-02-29

**Authors:** Sisi Yan, Huijuan Zhou, Shuiping Liu, Ji Wang, Yu Zeng, Froilan Bernard Matias, Lixin Wen

**Affiliations:** 1 Laboratory of Animal Clinical Toxicology, Department of Clinical Veterinary Medicine, College of Veterinary Medicine, Hunan Agricultural University, No. 1, Nongda Road, Changsha City, 410128 Hunan Province People’s Republic of China; 2 Changsha Lvye Biotechnology Co., Ltd, Changsha, Hunan Province People’s Republic of China; 3 Department of Animal Management, College of Veterinary Science and Medicine, Central Luzon State University, 3120 Science City of Muñoz, Nueva Ecija Philippines; 4 Hunan Collaborative Innovation Center of Animal Production Safety, No. 1, Nongda Road, Changsha City, 410128 Hunan Province People’s Republic of China

**Keywords:** Lard, Sunflower oil, Soybean oil, Obesity, Non-alcoholic fatty liver disease, Atherosclerosis

## Abstract

**Background:**

The traditional Chinese diet blends lard with vegetable oil, keeping the fatty acid balance intake ratio of saturated fatty acids, monounsaturated fatty acids, and polyunsaturated fatty acids at nearly 1:1:1. However, the effects of a mixture of lard and vegetable oil on lipid metabolism have never been researched. In the present study, by simulating Chinese high-fat dietary habits, we explored the effects of a mixture of lard and vegetable oil on lipid metabolism.

**Methods:**

We randomly assigned 50 male C57BL/6 J mice to 5 groups (10 in each group) and fed them lard, sunflower oil (SFO), soybean oil (SBO), lard blended with sunflower oil (L-SFO), or lard blended with soybean oil (L-SBO) for 12 weeks.

**Results:**

We found that the final body weights of mice in the lard group were significantly higher than those of mice in the SFO and SBO groups. Body fat rate and volume of fat cell of the lard group were significantly higher than those of the SFO, SBO, and L-SBO groups. Liver triglyceride level of the lard group increased significantly compared to the other groups. Although body fat rate and liver triglyceride level in the SBO and SFO groups decreased compared to those in the other groups, the high-density lipoprotein cholesterol/low-density lipoprotein cholesterol ratio were also significantly decreased in the SBO and SFO groups.

**Conclusions:**

We found that a lard diet induced accumulation of body fat, liver and serum lipids, which can increase the risk of obesity, non-alcoholic fatty acid liver disease, and atherosclerosis. The vegetable oil diet resulted in cholesterol metabolism disorders even though it did not lead to obesity. The mixed oil diet induced body fat accumulation, but did not cause lipid accumulation in the liver and serum. Thus, differential oil/fat diets have an impact on differential aspects in mouse lipid metabolism.

**Graphical abstract:**

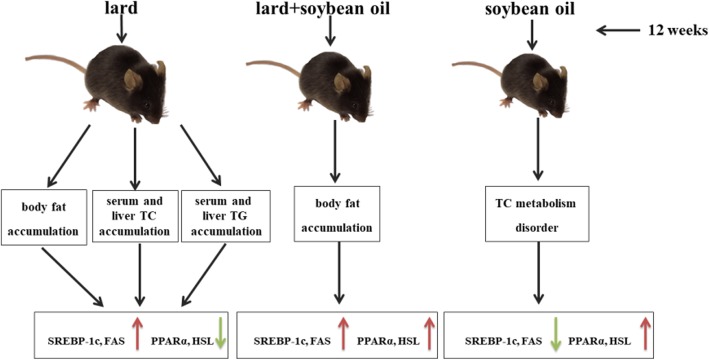

## Background

Obesity has become a public health concern worldwide. Obesity is highly associated with the development of hyperlipidemia, non-alcoholic fatty liver disease (NAFLD), and cardiovascular disease (CVD) [1]. Obesity leads to increased accumulation of free fatty acids (FFAs) and triacylglycerol (TG) in the serum, which are risk factors for the development of CVD [2]. Excessive TG accumulation in hepatocytes is a key feature in the development of NAFLD [3].

Western dietary habits typically involve high-fat consumption. Due to westernization over the past few years, the typical Chinese diet now also contains high fat [4, 5]. According to the Nutrition and Health Status of Chinese residents’ survey, the average daily intake of cooking oil or fat among Chinese residents were 42.1 g/day (37.3 g vegetable oil, 4.8 g lard) and 41.4 g/day (32.7 g vegetable oil and 8.7 g lard) in 2012 and 2002, respectively [6]. The Dietary Guidelines for Chinese residents (2016) indicate that more than 5% of Chinese residents have a daily consumption of cooking fat/oil that exceeds 95 g/day, with fat energy of diet up to 35~40% [7, 8]. Moreover, the intake of lard is decreasing due to negative reports concerning lard.

According to the World Health Organization (WHO), daily intake of energy obtained from fat/oil should be less than 30% and that from saturated fatty acids (SFAs) should be less than 10% [9].

The traditional Chinese diet blends lard with vegetable oil, which maintains the fatty acid balance intake ratio of SFAs, monounsaturated fatty acids (MUFAs), and polyunsaturated fatty acids (PUFAs) at nearly 1:1:1. However, the effect of mixing lard and vegetable oil on lipid metabolism has not been investigated. Previous research has focused on single oil/fat or oil mixtures containing either different vegetable oils or fatty acids [10, 11]. Vegetable oils rich in unsaturated fatty acids are usually regarded more beneficial than animal-derived fat rich in SFAs. Beef tallow diet reportedly led to greater body fat accumulation than olive oil and soybean oil (SBO) [12, 13]. Lard was reported to induce more body fat accumulation than safflower oil and linseed oil [14]. However, lard is often used in Chinese cooking [15, 16]. It was recorded that lard can relief liver poisoning according to the *Compendium of Material Medical*. The stereospecific position of fatty acid in lard is similar to milk fat, where palmitic acids are primarily in the sn-2 position, which benefits the absorption of Ca^2+^ [17]. Lard has higher content of α-tocotrienol than soybean oil, rice brain oil, and olive oil [18]. SFA diet can reduce competes with n-3 PUFA incorporation into tissue phospholipids compare to oleic diet [19]. Studies have found that soybean oil is more obesogenic than coconut oil rich in SFAs [20]. High fat diet with soybean oil induced high body weight more than high fat diet with palm oil and lard, which are both rich in SFAs [21]. In our previous study, traditional Chinese dietary habits of blending lard with SBO were proven to have anti-obesity effects when stimulated average oil intake of urban and rural residents in China [22]. This study aimed to investigate the effects of different fat/oil mixtures on lipid metabolism in mice when stimulated with typical Chinese residents’ high fat diet.

## Methods

### Animals, diets, and experimental design

Fifty male C57BL/6 J 6 weeks old mice were purchased from Hunan Silaike Laboratory Animal Co., Ltd. (Changsha, China). SBO and sunflower oil (SFO) were purchased from China Oil & Foodstuffs Co. Ltd. (Beijing, China),FuLinMen, and First Degree Press Oil. Leaf lard was purchased from a local supermarket, TangRenShen Co., Ltd. All mice were provided with food and water ad libitum and were kept under 12-h light-dark cycles at a temperature of 22 ± 1 °C and relative humidity of 65 ± 5%. After 1 week of acclimatization, the mice were randomly divided into five groups and fed different diets: lard, SFO, SBO, lard blended with SFO (L-SFO), and lard blended with SBO (L-SBO) for 12 weeks. The composition of the diets is shown in Table S1 while the fatty acid composition of the fat/oils is shown in Table S2. At the end of the feeding period, all mice were fasted for 12 h and anesthetized before being sacrificed. The blood and organs required for the study procedures were then collected.

### Sample collection and preparation

Blood samples were collected from the retro orbital plexus and were left standing overnight at 4 °C, The serum was isolated by centrifugation at 3500 g for 10 min at 4 °C and was immediately stored at − 80 °C until further analysis. Liver, epididymal adipose tissues, and perirenal adipose tissues were collected and weighed. Liver and epididymal adipose tissues were cut into five parts and washed with saline. One part was fixed in 10% neutral buffered formalin while the remaining parts were immediately frozen at − 80 °C until analysis.

### Measurements of lipid in plasma and liver

The levels of serum TG, total cholesterol (TC), high-density lipoprotein cholesterol (HDL-C), and low-density lipoprotein cholesterol (LDL-C) were measured using a Mindray Biochemical Analyzer BS-190 (Shenzhen, China). Serum FFAs, TG and TC were determined using an assay kit acquired from Nanjing Jiancheng Bioengineering Institute (Nanjing, China).

### Histological analysis

The epididymal white adipose tissues (WAT) and the left lateral lobe of the liver were fixed in 4% paraformaldehyde for 24 h. WAT was then stained with hematoxylin and eosin (H&E) and liver tissue was stained with Oil Red O (Sigma, USA). Stained areas were observed using an Olympus Photomicroscope (Olympus Inc., Tokyo, Japan) at a magnification of 400× for WAT and 200× for the liver tissue. The epididymal adipocyte area was measured using five fields of five individual fat cells, and epididymal adipocyte cross-section area (CSA) was calculated using Image-Pro Plus 5.1 (Media Cybernetics, Inc. Silver Spring, Maryland, USA). Liver Oil Red O-stained area was also measured using five fields of five individual samples in each group and was calculated using Image-Pro Plus 5.1.

### Western blotting analysis

The method of western blotting analysis of liver used was like that used in a previous study [22]. This method used antibodies including sterol regulatory-element binding proteins (SREBP)-1c (Biosynthesis Biotechnology Co., Ltd., Beijing, China), fatty acid synthase (FAS) (Epitomics, Inc. USA), peroxisome proliferator-activated receptor alpha (PPARα) (Epitomics, Inc. USA), hormone-sensitive lipase (HSL) (Santa Cruz, Inc. USA) glyceraldehyde 3-phosphate dehydrogenase (Proteintech, Inc. USA), and horseradish peroxidase-conjugated secondary antibodies (Proteintech, Inc. USA).

### Statistical analysis

The Feed efficiency ratio (FER) was computed by dividing the total weight gain (g) by the food intake (g) × 100. The collected dates were expressed as mean ± standard error of the mean (SEM). Mean differences between groups were analyzed using one-way analysis of variance (ANOVA) followed by least significant difference (LSD) post hoc analysis using SPSS 17.0 (SPSS Inc., Chicago, USA) software. A *P*-value < 0.05 was considered statistically significant. Graphical data presentations were created using Prism GraphPad version 5 (Graph Pad Software, San Diego, CA, USA).

## Results

### Body weight, feed efficiency ratio and body fat accumulation

**Fig. 1:**
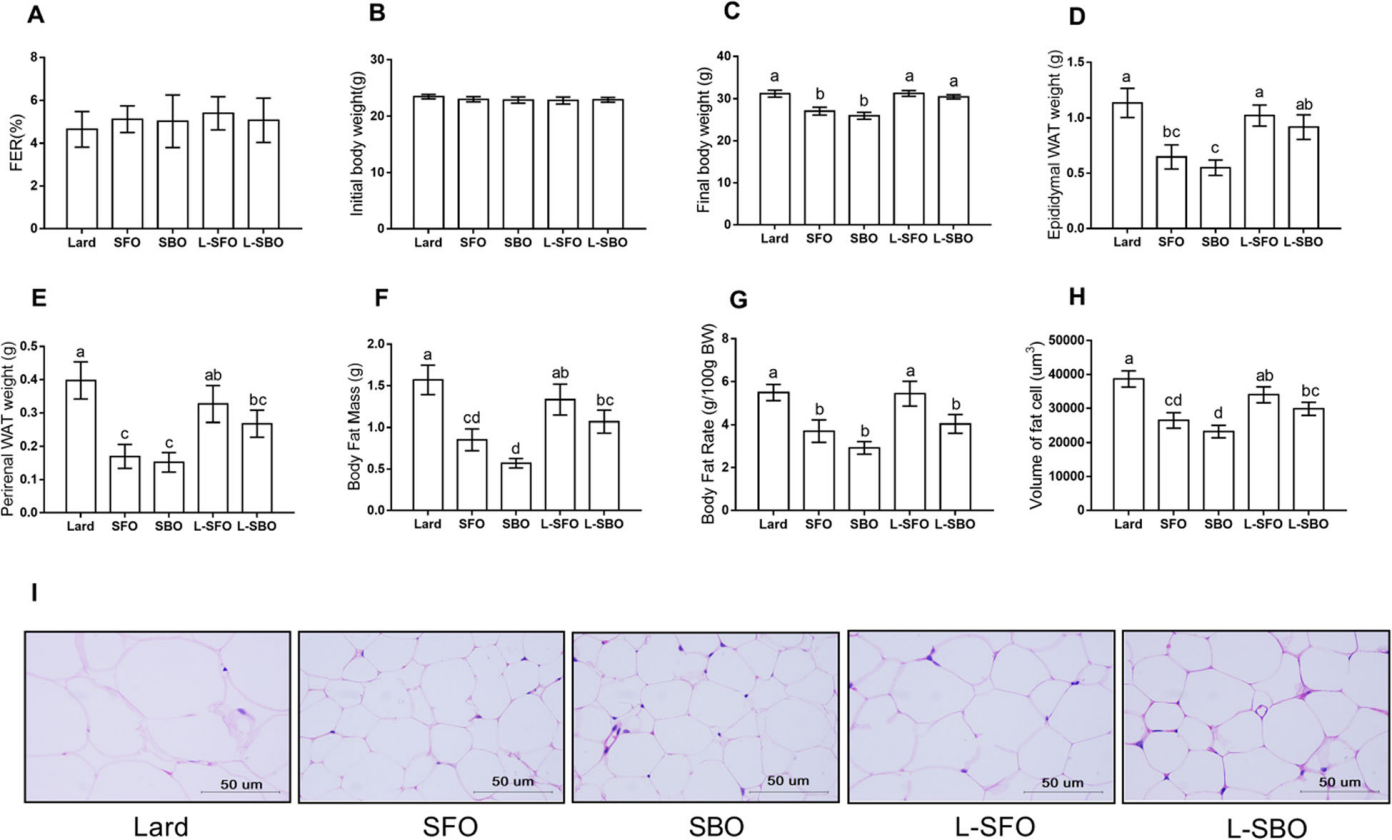
Effects of different dietary fat/oil on FER and body weight and body fat accumulation. Mice were fed different dietary fats/oils: lard, sunflower oil (SFO), soybean oil (SBO), lard blended with SFO (L-SFO), and lard blended with SBO (L-SBO). **a** Feed efficiency ratio (FER) = [weight gain (g)/food intake (g)] × 100; **b** initial body weight; **c** Final body weight; **d** epididymal white adipose tissue (WAT); **e** perirenal WAT; **f** body fat mass = epididymal WAT weight (g) + perirenal WAT weight (g); **g** body fat rate; =[epididymal WAT weight (g) + perirenal WAT weight (g)]/final body weight× 100; **h** cross-section area (CSA) of epididymal adipocyte; and (**i**) section of epididymal adipose tissue stained with **h** and **e**. Data were expressed as mean ± standard error of the mean, *n* = 9–10 per group except for (**a**), (**h**) and (**i**), *n* = 5 per group. Values with different superscript letters (a, b, c, and d) are significantly different at *P* < 0.05

There was no significant difference in the initial body weights between the groups (Fig. 1b). After 12 weeks of the experimental diet,the final body weights of the SFO and SBO groups were significantly lower than those in the lard group (Fig. 1c). The L-SFO and L-SBO groups showed a significantly higher final body weight compared to the SFO and SBO groups (Fig. 1c). However, the feed efficiency ratio did not differ between the groups (Fig. 1a). The intake of lard significantly increased the weight of the epididymal WAT, perirenal WAT, body fat mass and body fat rate compared to the intake of SFO and SBO (Fig. 1d-g). The SFO and SBO groups showed a significantly lower epididymal adipocyte CSA than the group fed with lard alone (Fig. 1h). The SFO and SBO groups showed a markedly lower epididymal adipocyte CSA than L-SFO and L-SBO groups (Fig. 1h).

### TC accumulation in the serum and liver

**Fig. 2:**
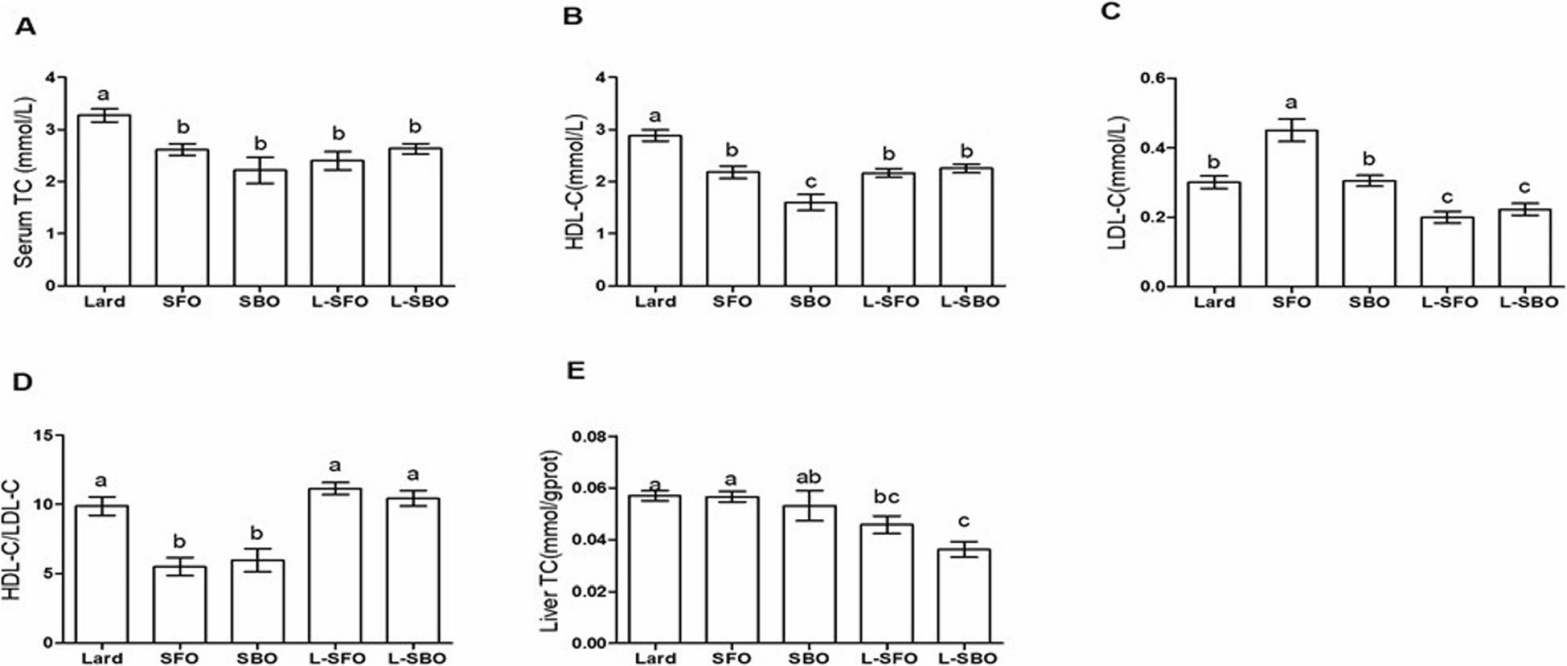
Effects of different dietary fat/oil on liver TC and serum TC, HDL-C and (LDL-C levels. Mice were fed different dietary fats/oils: lard, sunflower oil (SFO), soybean oil (SBO), lard blended with SFO (L-SFO), and lard blended with SBO (L-SBO). **a** Serum total cholesterol (TC); **b** HDL-C; **c** LDL-C; **d** HDL-C/LDL-C; and (**e**) liver TC. Data were expressed as mean ± standard error of the mean, *n* = 9–10 per group. Values with different superscript letters (a, b, c, and d) are significantly different at *P* < 0.05

The levels of serum TC and HDL-C were significantly lower in the L-SFO and L-SBO groups compared to the group fed with lard alone (Fig. 2a, b). When comparing the ‘mixed oil’ groups to the ‘vegetable oil’ groups, LDL-C serum levels were significantly lower in the L-SFO and L-SBO groups than those in the other three groups; however, no difference was observed when comparing theSFO and SBO groups with the lard group (Fig. 2c). These results indicate that the intake of an oil mixture could reduce levels of serum TC and LDL-C compared to the intake of lard alone. In addition, a noticeable decrease in TC level, as observed in the mice fed with vegetable oil, was mainly attributed to the reduced HDL-C level. Thus, the HDL-C/LDL-C ratio in the SFO and SBO groups were significantly lower than the other three groups (Fig. 2d). Liver TC levels in the L-SFO and L-SBO groups were also lower than those in the SFO and SBO groups (Fig. 2e).

### TG accumulation in the serum and liver

**Fig. 3:**
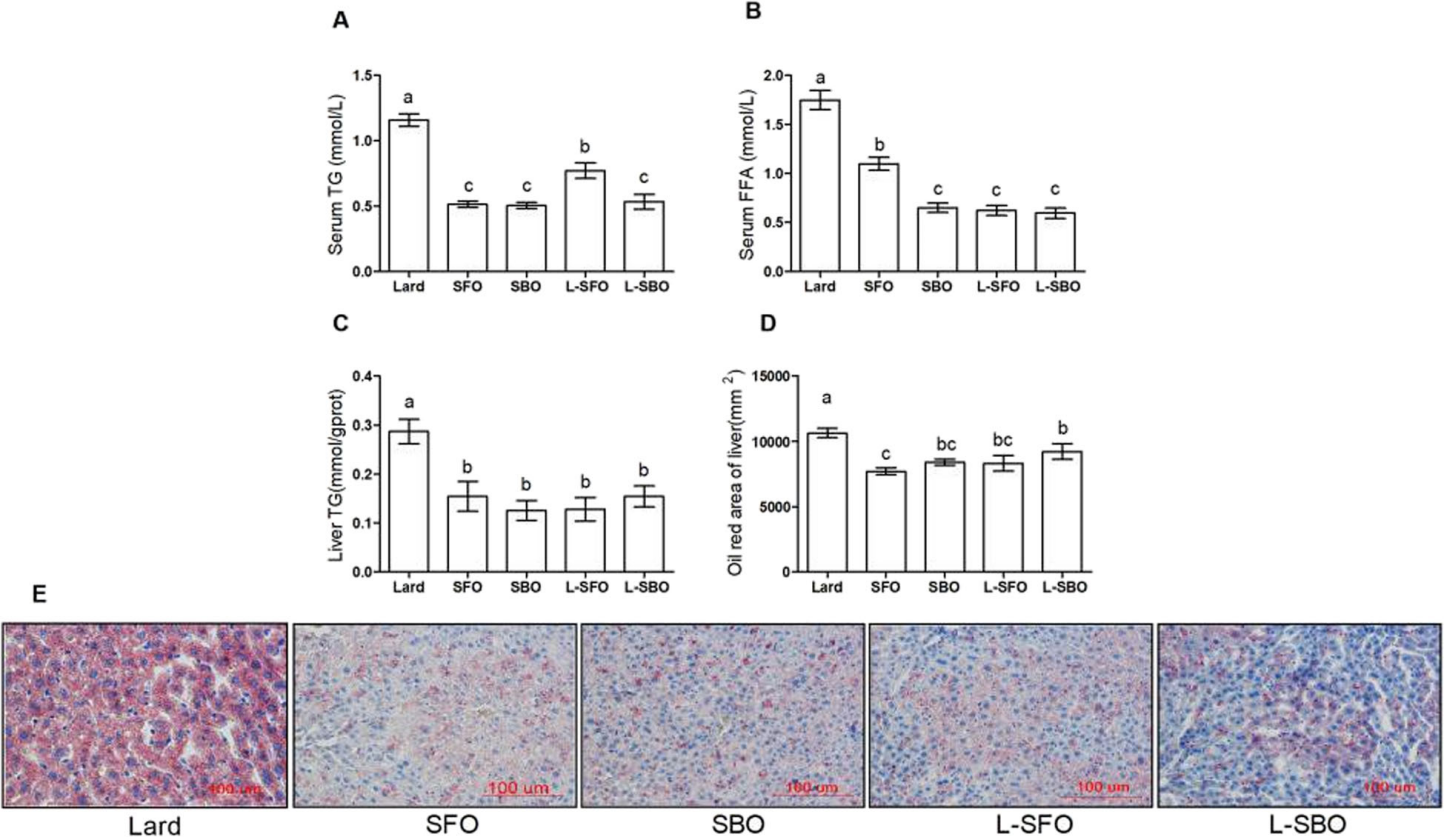
Effects of different fat/oil on TG and FFA in the serum and liver. Mice were fed different dietary fats/oils: lard, sunflower oil (SFO), soybean oil (SBO), lard blended with SFO (L-SFO), and lard blended with SBO (L-SBO). **a** Serum triacylglycerol (TG); **b** serum free fatty acids (FFAs); **c** liver TG; **d** Oil Red O area of the liver; and (**e**) sections of the liver stained with Oil Red O. Data were expressed as mean ± standard error of the mean, *n* = 8–10 per group except for (**d**) and (**e**), *n* = 5 per group. Values with different superscript letters (a, b, c, and d) are significantly different at *P* < 0.05

Levels of serum TG, FFA, and liver TG in the group fed with lard alone were markedly higher than those in the other four groups, indicating that a lard diet could result in TG accumulation both in the serum and liver (Fig. 3a-c). No significant difference was observed in the liver TG values between the SFO, SBO, L-SFO, and L-SBO groups (Fig. 3c). Oil Red O staining result verified the TG content of the liver (Fig. 3d). Thus, our results demonstrated that a mixed oil diet does not cause lipid accumulation in the serum and liver despite increasing the body weight.

### Expression of related proteins in the liver of mice fed experimental diets

**Fig. 4:**
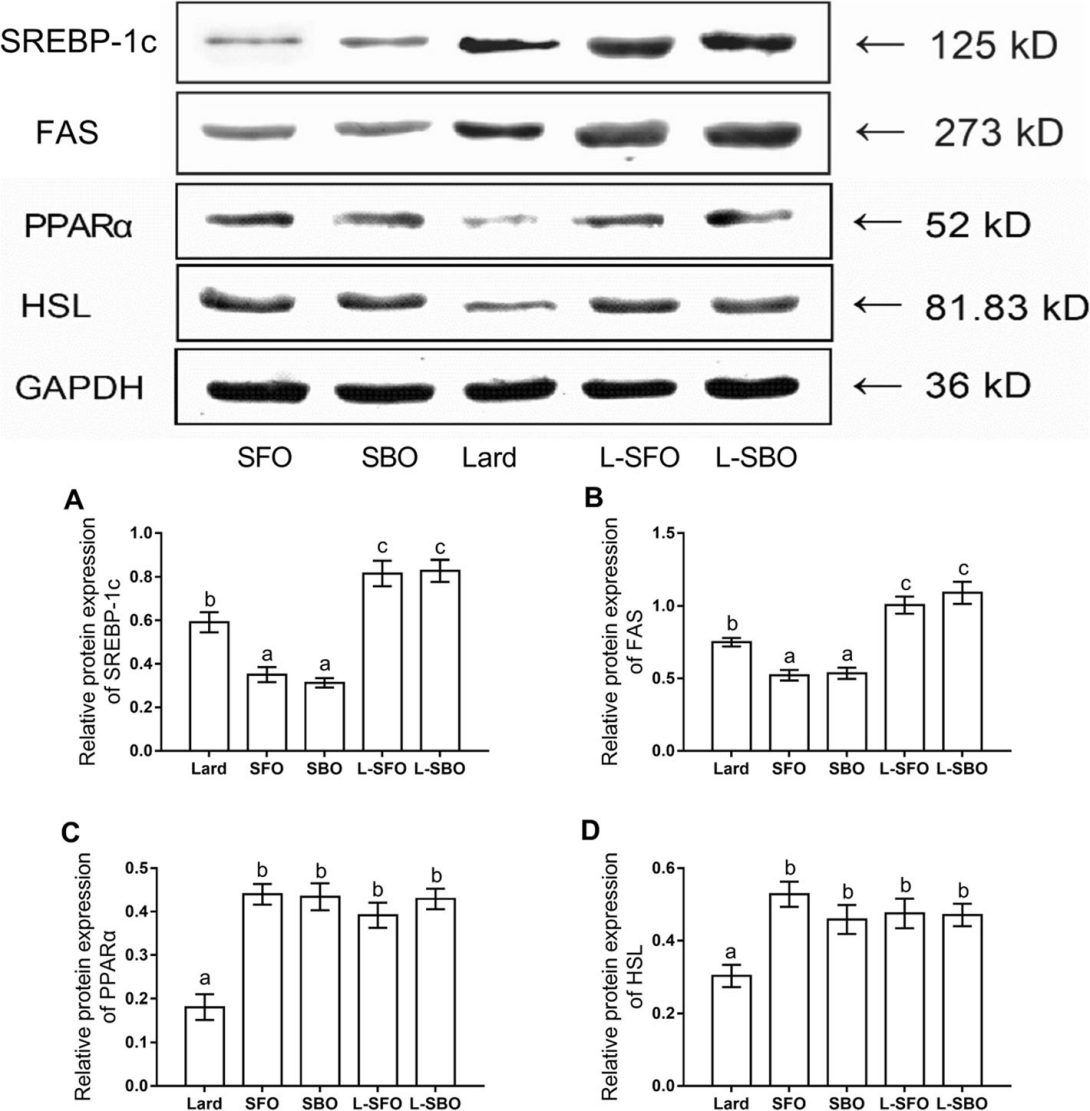
Effects of different fats/oils on (**a**) sterol regulatory-element binding protein (SREBP)-1c, (**b**) fatty acid synthase (FAS), (**c**) peroxisome proliferator-activated receptor alpha (PPARα) and (**d**) hormone-sensitive lipase (HSL) protein expression in the liver. Mice were fed different dietary fats/oils: Lard, sunflower oil (SFO), soybean oil (SBO), lard blended with SFO (L-SFO), and lard blended with SBO (L-SBO). Data were expressed as mean ± standard error of the mean, *n* = 3 per group. Values with different superscript letters (a, b, c, d) are significantly different at a *P* value < 0.05

Compared to the lard diet, the mixed oil diet increased expression of the SREBP-1c and FAS proteins, while simultaneously up-regulating the PPARα and HSL protein expression. Compared to the lard diet, the vegetable oil diet down-regulated expression of the SREBP-1c and FAS proteins and increased expression of the PPARα and HSL proteins. These findings illustrate that fatty acid synthesis was inhibited and hydrolysis of TGs was promoted by vegetable oil, contributing to the lower lipid accumulation compared to the lard diet (Fig. 4).

## Discussion

In this study, by simulating Chinese high-fat dietary habits, we explored the effects of an oil mixture (lard and vegetable oil) on lipid metabolism in mice. Our results showed that the lard diet led to the highest fat mass, followed by the mixture of lard and vegetable oil, and then vegetable oil. On the other hand, the vegetable oil diet resulted in disorders of cholesterol metabolism even with the lowest fat mass.

Lard, which is rich in SFA easily results in fat accumulation compared to vegetable oils, such as SBO, SFO, and corn oil [23–26]. This was verified in both our study and other studies. The ability to store fat may be more related to the source of dietary fat than to the total caloric intake [27]. SFA is a contributing factor to obesity; in the literature, edible beef tallow, which is rich in SFA, resulted in a larger amount of body fat accumulation than safflower oil, which is rich in n-6 fatty acid [28]. Body fat accumulation in SFA-rich diets is caused by lower oxygen consumption and decreased thermogenesis. SFA-rich diets affect membrane fatty acid composition. The metabolic rate is altered and in conjunction with the modification of membrane phospholipids, which induces a decrease in metabolic rate [29]. In addition, high lard diet (45% fat energy) was reported to up-regulated the expression of interleukin-6 and monocyte chemoattractant protein-1 in the retroperitoneal adipose tissue of mice, which promoted the development of inflammation that contribute to obesity [30, 31]. The palmitic acid in lard distributed in the Sn-2 position of the TG, making palmitic acid in lard more easily absorbable [32]. To sum up, it was inferred that palmitic acid, a source of SFA and rich in lard, may contribute to fat accumulation.

However, our results in this study conflict our previous research findings [22]. This may be due to differences in fat energy, as our previous study supplied 25% fat energy compared to 35% fat energy that was supplied in the present study. In general, a fat energy composition of up to 50–60% is observed in a high fat diet mouse model. Most researchers use these values to establish an obesity model [33] or a diabetic model [34]. According to Catta-Preta et al. [23], in a 60% fat energy diet (lard, olive oil, SFO, and canola oil separately), only lard contributes to fat mass (10% fat energy); In our study, mice were supplied with 35% fat energy are consistent with this report. Bargut et al. showed that the body fat mass of mice varied if the mice were fed different types of high-fat diets (50% fat energy), with the highest body fat mass being gained from lard and the lowest from fish oil [35]. Basically, essential nutrients should be consumed above a minimal level to avoid deficiency and below a maximal level to avoid toxicity. A U-shaped association is logical between nutrients and health. However, an extreme intake of oil is always applied in research when assessing its health effect [36].

Body fat accumulation rate in the L-SBO group was lower than in the L-SFO group. The proportion of n-3/n-6 PUFAs is an important factor in lipid metabolism. Studies have shown that a high n-3/n-6 PUFA ratio in dietary oil may improve the strength of oxidative stress through reductions in serum content of FFA [37]. The proportion of n-3/n-6 PUFA in L-SBO was higher than that in L-SFO.

In our study, HDL-C was lowest in mice fed with soybean oil. A randomized crossover studied two orally administered vitamin A-fat loads consisting of either 20% (wt:vol) soybean oil of 17% olive oil plus 3% soybean oil found that soybean oil induced postprandial decreases in HDL-C due to failed competition between soybean oil chylomicron remnants and HDL for hepatic lipase [38]. Besides, LDL-C was highest in mice fed with SFO and SBO. Mara et al. compared rats fed with cholesterol + olive oil or cholesterol + soybean oil and results showed that there was no significant difference in the final body weights of the groups, but the LDL-C level of rats fed with cholesterol + soybean oil was over 2 times higher than that of rats fed with cholesterol + olive oil [39]. In the present study, mice fed with SFO and SBO showed lowest HDL-C/LDL-C ratios, suggesting that SFO and SBO diets could lead to cholesterol disorders. However, a lack of initial HDL-C and LDL-C values and soya bean meal in fodder were limitations to support it. The proportion of MUFAs may be a factor that influences the metabolism of cholesterol. Duavy et al. (2017) showed that the intake of MUFA-rich olive oil reduced serum LDL-C levels compared to a SFO diet [39]. Although similar results were observed in the present study, the mechanisms underlying these results still need to be investigated further.

In this study, there was a significant increase in SREBP-1c in vegetable oil-fed mice. Tao Jiang et al. [40] found that SREBP-1c was up-regulated in mice that were fed lard with 60% fat energy, while in SREBP-1c knocked-out mice, renal lipid accumulation improved. SREBPs are the predominant isoforms expressed in most tissues and they control lipogenic gene expression [41]. Furthermore, they control the transcription of fatty acid synthase (FAS) which is a key component in the lipid synthesis pathway [42]. Endogenous fatty acids are mainly synthesized by FAS which synthesizes acetyl-CoA and malonyl-CoA into long-chain fatty acids [43]. These findings suggest that lard promotes the synthesis of fatty acids.

PPARα is a transcription factor that belongs to the nuclear hormone receptor superfamily and has been reported to induce expression of HSL and adipose triglyceride lipase, both of which contribute to the mobilization of TGs [44]. In the literature, hepatic PPARα protein increased in lard-fed mice [45]. However, there was a decrement in mice fed with lard compared to the other four groups; thus, HSL protein was lowest in mice fed with lard, indicating that lard hydrolysis capability was lowest.

Studies have shown that hypercholesterolemia is mainly caused by abnormally elevated levels of serum LDL-C [46]. High LDL-C and low HDL-C levels are associated with an increase in the risk of CVD [47]. The HDL-C/LDL-C ratio is an important indicator for the assessment of CVD risk and is more sensitive than TG and TC in predicting the risk of CVD. The HDL-C/LDL-C ratio of mice fed with vegetable oil was significantly lower than that of mice fed with oil mixture. These results indicate that the intake of vegetable oil increases the risk of CVD, compared to the intake of other oils. The proportion of MUFAs may be a factor that influences the metabolism of cholesterol. Duavy et al. (2017) showed that the intake of MUFA-rich olive oil reduced serum LDL-C levels compared to a SFO diet [48]. Although similar results were observed in the present study, the mechanisms underlying these results still need to be investigated further. The intake of lard lead to higher serum TG and FFA levels compared to the intake of vegetable oils in isolation or in an oil mixture. High serum TG and FFA levels increase the risk of atherosclerosis. This may be associated with high palmitic acid content at the Sn-2 position in lard which causes it to be directly absorbed from the intestine [49].

In the present study, the intake of lard enhanced fatty acid synthesis and attenuated mobilization of TG and compared to vegetable oil, contribute the highest fat accumulation. The oil mixture diet also enhanced fatty acid synthesis compared to vegetable oil; however, no differences in TG mobilization rate were observed between the mice that consumed the oil mixture and those that consumed the vegetable oil diets. This may be attributed to a lower liver TG content in the diet of the mice that were fed vegetable oil and oil mixture than those fed with lard.

However, this study only compared five types of oil diets, without a control group. Thus, we discussed the effects of different oil diets on lipids metabolism based on 35% fat energy consumption in the present study.

## Conclusion

Overall, after simulating high-fat dietary habits of Chinese residents, the intake of a mixture of lard and vegetable oil did not have anti-obesity effects compared to vegetable oils. In addition, we found that intake of lard induced body fat accumulation and lipid accumulation in the liver and serum and increased risk of obesity and atherosclerosis. Intake of vegetable oil resulted in disorders pertaining to cholesterol metabolism, which advanced the risk of CVD even though it did not lead to obesity. Intake of oil mixture, despite not resulting in lipid accumulation in the liver and serum, inevitably induced body fat accumulation. Thus, differential oil/fat diets have an impact on differential aspect in mice lipid metabolism.

## Supplementary information

**Additional file 1: Table S1.** Composition of the diets (g/kg). **Table S2.** Fatty acids composition of the fat/oils

## Data Availability

All data generated or analyzed are included in this paper.

## Abbreviations

CSA: Cross-section area
FAS: Fatty acid synthase
FER: Feed efficiency ratio
FFA: Free fatty acid
H&E: Hematoxylin and eosin
HDL-C: High-density lipoprotein cholesterol
HSL: Hormone-sensitive lipase
LDL-C: Low-density lipoprotein cholesterol
L-SFO: Blended lard and sunflower oil
L-SBO: Blended lard and soybean oil
MUFA: Monounsaturated fatty acid
PPARα: Peroxisome proliferator-activated receptor alpha
PUFA: Polyunsaturated fatty acid
SBO: Soybean oil
SFA: Saturated fatty acids
SFO: Sunflower oil
SRE: Sterol regulatory-element
SREBP: Sterol regulatory-element binding protein
TBST: Tris-buffered saline and Polysorbate 20
TC: Total cholesterol
TG: Triglyceride
WAT: White adipose tissue
