# Supplementary material for: Differential effects of Chinese high-fat dietary habits on lipid metabolism: mechanisms and health implications
Source: Lipids Health Dis. 2020 Feb 29;19:30. doi: 10.1186/s12944-020-01212-y (PMC7049192; doi:10.1186/s12944-020-01212-y)
Supplement: Supplementary file 1 — Additional file 1: Table S1. Composition of the diets (g/kg). Table S2. Fatty acids composition of the fat/oils [file 12944_2020_1212_MOESM1_ESM.docx]

| Ingredient | Lard | SFO | SBO | L-SFO | L-SBO |
| --- | --- | --- | --- | --- | --- |
| Corn starch | 275 | 275 | 275 | 275 | 275 |
| Wheat bran | 280 | 280 | 280 | 280 | 280 |
| Soybean meal | 180 | 180 | 180 | 180 | 180 |
| Fish meal | 100 | 100 | 100 | 100 | 100 |
| Beer yeast | 20 | 20 | 20 | 20 | 20 |
| Premix | 40 | 40 | 40 | 40 | 40 |
| Sunflower oil | 0 | 105 | 0 | 57.7 | 0 |
| Soybean oil | 0 | 0 | 105 | 0 | 56.7 |
| Lard oil | 105 | 0 | 0 | 47.3 | 48.3 |
| Total energy (kcal/kg) | 3730 | 3730 | 3730 | 3730 | 3730 |
| Energy from lipid (%) | 35 | 35 | 35 | 35 | 35 |

Table S1: Composition of the diets (g/kg)

The diet ingredients were prepared according to China’s Laboratory Animals Nutrients for Formula Feeds [GB 14924.3-2010]

Table S2: Fatty acids composition of the fat/oils

|  | Lard | SFO | L-SFO | SBO | L-SBO |
| --- | --- | --- | --- | --- | --- |
| Fatty acids | % total fatty acids | | | | |
| C4:0 |  |  |  |  |  |
| C6:0 |  |  |  |  |  |
| C8:0 |  |  |  |  |  |
| C10:0 | 0.08 |  | 0.04 |  | 0.04 |
| C11:0 |  |  |  |  |  |
| C12:0 | 0.07 |  | 0.04 |  | 0.03 |
| C13:0 |  |  |  |  |  |
| C14:0 | 1.49 | 0.08 | 0.86 | 0.08 | 0.73 |
| C14:1 |  |  |  |  |  |
| C15:0 | 0.06 |  | 0.03 |  | 0.03 |
| C15:1 |  |  |  |  |  |
| C16:0 | 28.80 | 6.55 | 18.79 | 10.80 | 19.08 |
| C16:1 | 1.52 | 0.12 | 0.89 | 0.09 | 0.75 |
| C17:0 | 0.83 | 0.09 | 0.50 | 0.19 | 0.48 |
| C17:1 |  |  |  |  |  |
| C18:0 | 20.50 | 3.15 | 12.69 | 3.94 | 11.56 |
| C18:1n9t | 0.11 |  | 0.06 |  | 0.05 |
| C18:1n9c | 33.60 | 27.20 | 30.72 | 23.80 | 28.31 |
| C18:2n6t |  |  |  |  |  |
| C18:2n6c | 10.90 | 61.50 | 33.67 | 53.20 | 33.74 |
| C20:0 | 0.27 | 0.23 | 0.25 | 0.37 | 0.32 |
| C18:3n6 |  |  |  |  |  |
| C20:1 | 0.68 | 0.15 | 0.44 | 0.69 | 0.69 |
| C18:3n3 | 0.49 | 0.07 | 0.30 | 6.25 | 3.60 |
| C21:0 |  |  |  |  |  |
| C20:2 | 0.45 |  | 0.25 |  | 0.21 |
| C22:0 |  | 0.67 | 0.30 | 0.43 | 0.23 |
| C20:3n6 |  |  |  |  |  |
| C22:1n9 |  |  |  | 0.10 | 0.05 |
| C20:3n3 |  |  |  |  |  |
| C20:4n6 | 0.22 |  | 0.12 |  | 0.10 |
| C23:0 |  |  |  |  |  |
| C22:2 |  |  |  |  |  |
| C24:0 |  | 0.15 | 0.07 | 0.09 | 0.05 |
| C20:5n3 |  |  |  |  |  |
| C24:1 |  |  |  |  |  |
| C22:6n3 |  |  |  |  |  |
| SFA | 52.10 | 10.90 | 33.57 | 15.90 | 32.60 |
| MUFA | 35.80 | 27.47 | 32.05 | 24.58 | 29.74 |
| PUFA | 11.61 | 61.57 | 34.09 | 59.45 | 37.44 |
| n6/n3 | 22.69 | 878.57 | 40.41 | 8.51 | 9.40 |

1. The fatty acid profiles using [Agilent](http://www.baidu.com/baidu.php?url=K600000F3Z4zzZ2Y9ymsDpOY36UafjTdAIwECY7QJWszK0fWTiCKXPMltqrAwqkMlwf1eXLZjZiiERDJ6UGIpkzFRVpWp72aM-eEfiuZd2o3jQTLqVIgtR4m7o1naZmO0lgmbKvhw50BJemRYlko4f_rlA_5vMZbRJmwjpzV-I5fjpUE8AHAqk8TAFl6MUGi_Xxevn4P6I5gZuN9j0.7D_aJQQICqB7BmLIPMHuuvUrg_4TXGmuCyrhz1xER0.U1Yk0ZDq_Q2qLTQg0A7bTgbq_Q2qLTQg0A7bTgfqn6KspynqnfKY5y7Mpyk-UMis_oLIsVT0pyYqnWcd0ATqIyNsT100Iybqmh7GuZN_UfKspyfqnHT0mv-b5Hndn6KVIjYknjD4g1DsnHIxn1msnfKopHYs0ZFY5HDdrfK-pyfqnHf1PdtznHDkPdtzrjTLPNtzrjTzP7tzrjRdr7tzrjR3P7tzrjmsP7tzrjmzPdtznjTkr0KBpHYznjwxnHRd0AdW5HDsnj7xrjD3rjR4nH6Yg100TgKGujYs0Z7Wpyfqn0KzuLw9u1Ys0A7B5HKxn0K-ThTqn0KsTjYs0A4vTjYsQW0snj0snj0s0AdYTjYs0AwbUL0qnfKzpWYs0Aw-IWdsmsKhIjYs0ZKC5H00ULnqn0KBI1Ykn0K8IjYs0ZPl5fK9TdqGuAnqTZnVmvY0IZN15HDLP16dPWcknHDYrjfLPHbYrjfL0ZF-TgfqnHRdn1TvPjR3Pjn3n6K1pyfquyP-nvR1njTsnj0snHD4ufKWTvYqrDFKn1bsrjnsP1wAPHc3f6K9m1Yk0ZK85H00TydY5H00Tyd15H00XMfqn0KVmdqhThqV5HKxn7ts0Aw9UMNBuNqsUA78pyw15HKxn7ts0ZK9I7qhUA7M5H00uAPGujYknjT1P1fkrjcv0ANYpyfqQHD0mgPsmvnqn0KdTA-8mvnqn0KkUymqnHm0uhPdIjYs0AulpjYs0Au9IjYs0ZGsUZN15H00mywhUA7M5HD0UAuW5H00ULfqn0KETMKY5H0WnanWnans0APzm1Y1P1D1&word=%E5%AE%89%E6%8D%B7%E4%BC%A6&ck=0.0.0.0.0.0.0.0&shh=www.baidu.com&sht=56060048_4_pg) Gas Chromatograph 7890A according to GB 5009.168-2016, fatty acid test in food.
2. SFA: saturated fatty acid; MUFA: monounsaturated fatty acid; PUFA: polyunsaturated fatty acid
